# Evaluation and correlation of oral cancer phobia and anxiety in oral submucous fibrosis patients: A cross-sectional study

**DOI:** 10.1016/j.jobcr.2025.04.008

**Published:** 2025-04-23

**Authors:** Ashita Ritesh Kalaskar, Gauri Ashok Somani, Ritesh Rambharose Kalaskar

**Affiliations:** a Department of Oral Medicine & Radiology, Government Dental College & Hospital, Nagpur, 440003, Maharashtra, India; b Government Dental College & Hospital, Nagpur, 440003, Maharashtra, India; c Department of Pediatric Dentistry, Government Dental College & Hospital, Nagpur, 440003, Maharashtra, India

**Keywords:** Anxiety, Phobia, Oral cancer, Oral submucous fibrosis

## Abstract

**Objective:**

Oral Cancer Phobia and anxiety in patients having premalignant conditions like Oral Submucous fibrosis (OSMF) needs to be evaluated so as to provide an overall effective management to these patients.

**Methods & materials:**

Patients having OSMF were tested for oral cancer phobia and anxiety using the validated Marathi translation of CAI and 5-item version of the Spielberger State-Trait Anxiety Inventory (STAI) questionnaire respectively. The correlation was obtained for Oral cancer phobia, anxiety, and socio-demographic status.

**Results:**

Cancer phobia and anxiety (mild to severe) were present in all the participants with the majority (65 %) having severe scores and significantly correlated with each other (p value < 0.001). Their scores significantly increased with an increase in grades of OSMF. A co-relation with socio-demographic profiles revealed females to be more affected by severe cancerophobia and anxiety. Patients with less education and housewives had greater levels of cancerophobia and anxiety. Housewives were also more concerned about acquiring cancer.

**Conclusion:**

Cancer phobia and anxiety are prevalent in Oral Submucous Fibrosis patients highlighting the significance of identifying and providing psychological therapy as an important part of OSMF management protocol.

## 1 Introduction

Oral Submucous Fibrosis (OSMF) is a chronic, progressive, potentially malignant condition of the oral cavity that predominantly affects people of South-East Asian Origin. Recent data elucidate that cases have increased dramatically from an estimated 250,000 cases in 1980 to 14 million cases in 2010.^1^ As per the Indian context, the reported prevalence of OSMF is 6.42 per 1000 cases having male to female ratio of 4.9:1 and the malignant transformation to be 2.3–7.6 %.^2^^,^^3^ These patients suffer from a progressive reduction of mouth opening with complications like difficulty in deglutition, speaking, and hearing impairment. Although many treatment options (medicinal, surgical and physiotherapy) have been tried but till date complete relief has not been reported.

Being a chronic premalignant condition with debilitating consequences, the patients could be associated with psychiatric morbidities. Mubeen K et al.,^4^ Raja JV et al.,^5^ and Arjun TN et al.^6^ have reported anxiety to be associated with OSMF which increases as the stage advances. With the advancement of technology and media, awareness regarding oral cancer has increased a lot, the negative impact of which could lead to attitudinal changes in OSMF patients. Fear of oral cancer in these patients can either lead to refraining oneself from screening or frequent unwanted visits to different oral physicians or affect the treatment outcome like unresponsiveness to any therapy. Such attitudinal changes in these patients needs to be identified and understood which could further help in exploring and improving prevention and management strategies inclusive of appropriate behavioural therapy. Thus, this study was planned to evaluate the prevalence of oral cancer phobia and anxiety in patients having OSMF and explore their correlation with disease severity and socio-demographic factors to improve patient management.

## 2 Methods

The present observational cross-sectional study was conducted on Oral Submucous Fibrosis patients of age 18 years and above visiting dental OPD in Oral Medicine and Radiology Department, from August 2022 to October 2022. The protocol was approved by the institutional ethics committee (IEC/05/18, dated: April 20, 2022). Depending on the approximate number of OSMF patients reporting to OPD in one and half month (200), margin of error 5 %, and confidence level of 95 %, the sample size was calculated from an online calculator (http://www.raosoft.com/samplesize.html) using formula n = N∗X/(X + N = 1). The sample size calculated was 132 and after considering 10 % non-responsive rate, it was calculated as 150 OSMF patients.

OSMF Patients with a history of psychiatric disorders, coexisting systemic diseases (cardiovascular diseases, asthma, chronic obstructive pulmonary disease, diabetes mellitus, arthritis, carcinoma, migraine, and HIV infection), other oral mucosal disorders (burning mouth syndrome, oral lichen planus, and recurrent aphthous stomatitis), miscellaneous diseases (temporomandibular joint disorders, facial neuralgia, atypical facial pain, atypical odontalgia, bruxism, salivary gland diseases, chronic advanced periodontitis, and viral infection), premalignant or malignant lesions during the study period or in the past (Group IV B OSMF patients as per Khanna JN and Andrade NN 1995 classification),^7^ pregnant females and the participants not willing to participate in the study were excluded from the study.

OSMF patients aged 18 years and above, diagnosed clinically (group I,II III and IV A as per Khanna JN and Andrade NN 1995 classification),^7^ consenting to participate in the study and able to understand Marathi, were included in the study. They were further evaluated for the following: socio-demographic data which included age categories as 18–40, 41–60 and above 60 years), gender (male and female), education (Higher Secondary School, Graduate, Higher Education), marital status (reported in 5 categories ’married/living as married’, ‘divorced’, ‘separated’, ‘widowed’, ‘single’, and dichotomized into ‘married or cohabiting’ and ‘not married or cohabiting’) and occupation (professional, semi-professional, house wives).

Validated Marathi translation of the Cancer Attitude Inventory (CAI) was used to evaluate oral cancer phobia which contains 39- items measuring attitudes towards cancer that encompasses a range of domains including – cancer stigma, economic hardship, and potential for positive growth which represents different aspects of cancer fear.^8^^,^^9^ It uses a 5-point Likert scale with responses as don't know, never, occasionally, fairly often, and very often (from 0 to 4 points respectively). Scoring for cancer phobia was No Phobia:0–37, Mild Phobia: 38–74, Moderate Phobia:75–111, and Severe Phobia:112–148 ^8,9^. For evaluating Anxiety**:** 5-item version of Spielberger State- Trait Anxiety Inventory (STAI) was used.^10^ It measures state and trait anxiety using a 4-point Likert-type scale with responses as not at all, somewhat, moderately so and very much so (from 1 to 4 points respectively). The scoring for anxiety was No anxiety = 0–1,Mild anxiety = 6–10, Moderate anxiety = 11–15 and Severe anxiety = 16–20 ^10^. After receiving patients responses, the data was analysed, scored and tabulated for statistical analysis.

### 2.1 Statistical tools

Data collected was compiled into a MS Office excel worksheet & was subjected to statistical analysis using SPSS software version 20.0. Chi-square test was used to find out the association of oral cancer phobia & socio-economic & other related factors. Pearson's correlation test was used to assess correlation of OSMF stages with CAI and STAI score. P < 0.05 was considered statistically significant.

## 3 Results

A total of 163 patients with different grades of OSMF were included in the final sample. There were 125 males and 38 females, with an average age of 35 (±10.9) years, ranging from 18 to 67 years. Majority of the patients (73 %) had arecanut with smokeless tobacco consumption habit. Over 43.6 % of the participants had education till higher secondary and graduation, about 52.8 % were semi-professionals in occupation, and 19 % were housewives (Table 1).

**Table 1:** Distribution of the socio-demographic characteristics of the participants.

Socio-Demographic Factors		Frequency (N = 163)	Percent (%)
**Age Groups**	Young	66	40.5
	Middle	66	40.5
	Old	31	19.0
**Gender**	Male	125	76.7
	Female	38	23.3
**Habits**	Only arecanut	15	9.2
	Arecanut with smokeless tobacco	120	73
	Arecanut with smoking tobacco	28	17.17
**Education**	Uneducated	65	39.9
	SSC	27	16.6
	HSC & Graduates	71	43.6
**Occupation**	Semi-Professionals	86	52.8
	Students	21	12.9
	Housewives	31	19.0
	Professionals	25	15.3

**Fig. 1:**
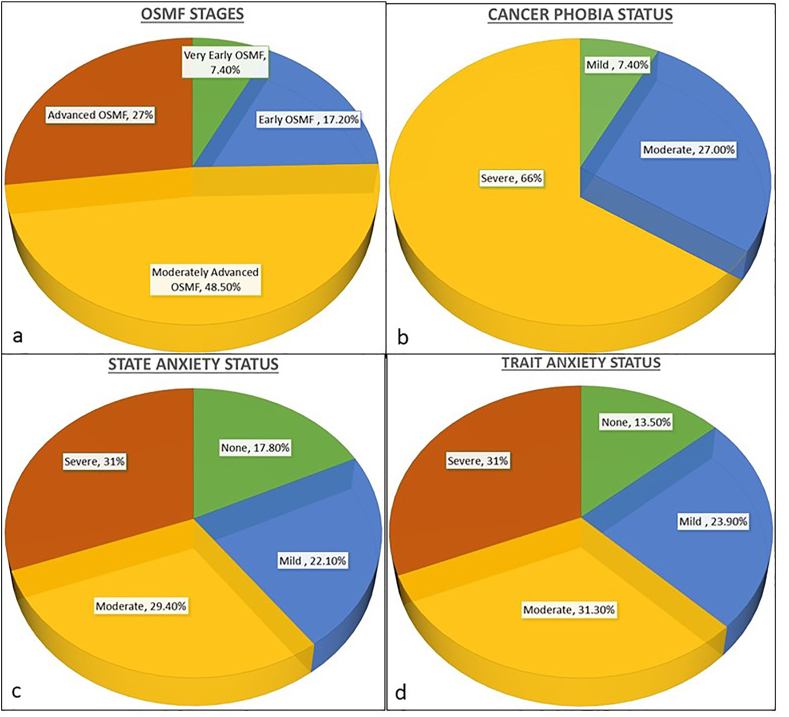
Distribution of Clinical & Psychological Status of the participants.

Clinically, over 75 % participants were diagnosed in moderate and advanced stages of OSMF. Almost all the patients had oral cancer phobia with majority of the participants (65 %) assessed having severe cancer phobia. Similarly, 82.50 % and 86.2 % patients had state and trait anxiety respectively, out of which over 60 % patients had moderate to severe state and trait anxiety of developing cancer (Fig. 1). The mean scores of cancer phobia and STAI were 123 (±29) and 12 (±5.5) respectively.

On comparing various socio-demographic characteristics with OSMF, about 50 % males and females, and 50 % young and middle-aged participants had moderately advanced stages of OSMF, while over 45 % old patients had advanced OSMF (Table 2). Similarly, most of the higher educated and semi-professionals had moderately advanced stages of OSMF. However, this association was not found to be statistically significant (p value > 0.05).

**Table 2:** Comparison of various socio-demographic characteristics with OSMF stages.

OSMF		Very early OSMF	Early OSMF	Moderately advanced OSMF	Advanced OSMF	Chi-Square (p value)
Total		12	28	79	44	
		7.4 %	17.2 %	48.5 %	27.0 %	
Gender	Male	11	25	60	29	0.076
		8.8 %	20.0 %	48.0 %	23.2 %	
	Female	1	3	19	15	
		2.6 %	7.9 %	50.0 %	39.5 %	
Age Group	Young	6	9	35	16	0.092
		9.1 %	13.6 %	53.0 %	24.2 %	
	Middle	3	13	36	14	
		4.5 %	19.7 %	54.5 %	21.2 %	
	Old	3	6	8	14	
		9.7 %	19.4 %	25.8 %	45.2 %	
Education	Uneducated	8	10	24	23	0.134
		12.3 %	15.4 %	36.9 %	35.4 %	
	SSC	1	5	16	5	
		3.7 %	18.5 %	59.3 %	18.5 %	
	HSC & Graduates	3	13	39	16	
		4.2 %	18.3 %	54.9 %	22.5 %	
Occupation	Semi-Professionals	9	17	42	18	0.334
		10.5 %	19.8 %	48.8 %	20.9 %	
	Students	2	4	11	4	
		9.5 %	19.0 %	52.4 %	19.0 %	
	Housewives	1	3	15	12	
		3.2 %	9.7 %	48.4 %	38.7 %	
	Professionals	0	4	11	10	
		0.0 %	16.0 %	44.0 %	40.0 %	

**Fig. 2:**
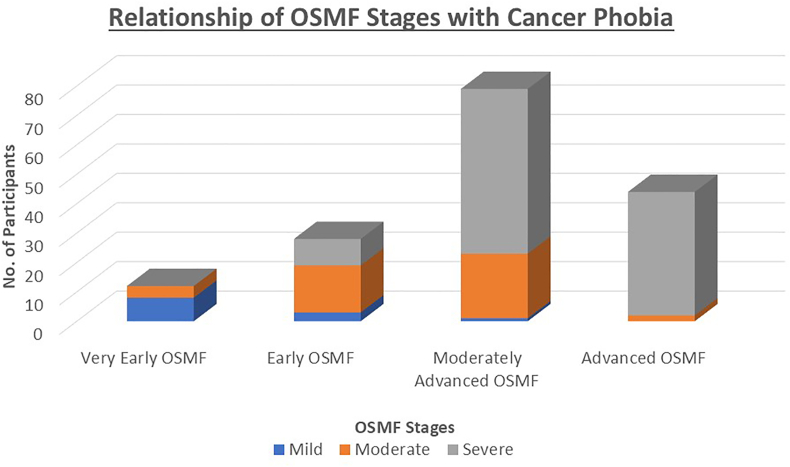
Relationship of OSMF stages with cancer phobia.

On comparing various characteristics with cancer phobia, about 80 % females and 60 % males, and majority of young, middle and old aged participants had severe phobia of developing cancer. Similarly, most lower-educated housewives had severe phobia (Table 3). However, this association was not found to be statistically significant (p value > 0.05). Early stages of OSMF were related to milder phobia, while moderately advanced and advanced OSMF condition was associated with more fear of cancer development among participants. This association was found to be statistically significant (p value < 0.001) (Table 3, Fig. 2).

**Table 3:** Comparison of various socio-demographic characteristics with Cancer Phobia Status.

Cancer Phobia Status		Mild Phobia	Early Phobia	Severe Phobia	Chi-Square (p value)
Total		12	44	107	
		7.4 %	27.0 %	65.6 %	
Gender	Male	10	38	77	0.14
		8.0 %	30.4 %	61.6 %	
	Female	2	6	30	
		5.3 %	15.8 %	78.9 %	
Age Group	Young	5	17	44	0.886
		7.6 %	25.8 %	66.7 %	
	Middle	6	18	42	
		9.1 %	27.3 %	63.6 %	
	Old	1	9	21	
		3.2 %	29.0 %	67.7 %	
Education	Uneducated	8	14	43	0.173
		12.3 %	21.5 %	66.2 %	
	SSC	0	7	20	
		0.0 %	25.9 %	74.1 %	
	HSC & Graduates	4	23	44	
		5.6 %	32.4 %	62.0 %	
Occupation	Semi-Professionals	6	26	54	0.414
		7.0 %	30.2 %	62.8 %	
	Students	3	6	12	
		14.3 %	28.6 %	57.1 %	
	Housewives	2	4	25	
		6.5 %	12.9 %	80.6 %	
	Professionals	1	8	16	
		4.0 %	32.0 %	64.0 %	
OSMF	Very early OSMF	8	4	0	<0.001∗∗
		66.7 %	33.3 %	0.0 %	
	Early OSMF	3	16	9	
		10.7 %	57.1 %	32.1 %	
	Moderately advanced OSMF	1	22	56	
		1.3 %	27.8 %	70.9 %	
	Advanced OSMF	0	2	42	
		0.0 %	4.5 %	95.5 %

On comparing various characteristics with state and trait anxiety, it was found that most of the males had mild or moderate anxiety and over 60 % females had severe anxiety. Patients with lower education reported higher trait anxiety. Likewise, housewives were more anxious of developing cancer. These findings were found to be statistically significant. The state and trait anxiety scores also increased with the rising stages of OSMF among participants. (p value < 0.001). For the continuous variables, 95 % confidence interval (95 % CI) has been provided (Table 4).

**Table 4:** Confidence interval (CI) for continuous variables.

	N	Minimum	Maximum	Mean	Std. Deviation	95 %CI	
						LOWER	UPPER
CancerPhobia_score	163	50	178	122.81	28.688	118.374	127.24
StateAnxiety_score	163	5	20	12.46	5.445	11.61	13.30
TraitAnxiety_score	163	5	20	12.91	5.285	12.09	13.72
Valid N (listwise)	163						

Patients with severe cancer phobia had statistically significant (p-value<0.01) severe state and trait anxiety scores and their corelation was also found to be statistically significant.

## 4 Discussion

Around three quarters of the patients in our study were males. Similar findings have also been reported by Srivastava R et al.,^11^ and Sowmya S et al..^12^ Sumithrarachchi S et al.^13^ reported higher tobacco (93.2 %) and areca-nut (79.6 %) use among males. Thus higher occurrence of OSMF in males could be attributed due to their frequent consumption of tobacco and arecanut. This is also supported by the findings of high percentage (73 %) of OSMF patients having habit of arecanut with smokeless tobacco in the present study.

Both genders and all age groups had higher phobia of developing cancer. Similarly, low-educated patients and housewives reported higher cancer phobia. Awareness of health hazards due to consumption of tobacco, betelnut, kharra etc might seem to serve as a double-edged sword. Although various awareness modalities are trying to educate and prevent people from consuming these harmful products, some strata of the population can develop psychological problems like anxiety and cancer phobia. These are the vulnerable people who need additional attention while planning their management protocol. The findings of study done by Bahrami F et al.^14^ have reported that females are more prone to anxiety than males because of their thought control strategies and metacognitive beliefs. Females have metaworry more than males because metacognitive beliefs about uncontrollability of worry are more prevalent in females. It means that females believe that worry is uncontrollable. As a result, they worry about their worry and suffer from type II worry.^14^ Likewise, the present study demonstrates that women are prone to possess phobia and anxiety. This explains the reason for severe phobia demonstrated by females and housewives as compared to males and other professionals respectively, although the results were non-significant.

This study has reported a higher occurrence of cancer phobia and anxiety among OSMF patients, the severity of which increases with the increasing severity of the disease. Higher anxiety levels with higher stages of OSMF have also been highlighted by other studies.^4^^,^^15^, ^16^, ^17^ Mubeen K et al.^4^ had compared psychological morbidity using General Health Questionnaire (GHQ-28), among clinically diagnosed OSMF patients and controls, wherein GHQ scores were the highest among the worst functional stages of OSMF. Gondivkar SM et al.^16^ had found OSF patients with lower Socioeconomic Status had worse OHRQoL as compared with those with middle and upper socioeconomic status. Raja JV et al.^5^ found substantial psychiatric morbidity among patients with OSMF, compared to areca nut chewers without OSMF and patients with other dental problems.

Chaudhary et al.^18^ systematically reviewed the literature of over hundred years regarding Quality of Life (QoL) in OSMF, and concluded that OSMF not only physically debilitates a patient, but has repercussions on the social, physical, psychological domains as well. Apart from trismus, ulcerations, burning sensation and worsening of dental health, this pre-malignant condition causes much psychological burden to patient's QoL.^18^ Thus as the severity of the symptoms increases, there is a surge in alterations in all the three domains of social, physical and psychological.

With respect to all these, oral cancer phobia i.e. a constant fear of having or converting to oral cancer was not evaluated specifically in OSMF patients. Oral Cancer Phobia can be an important contributory factor towards the worsening psychological burden in these patients which can be a hindrance in achieving a successful treatment outcome. Thus early identification of anxiety and oral cancer phobia in OSMF patients and adequate counselling in this aspect in addition to the therapeutic protocol should always be considered. Also, regular awareness and counselling campaigns should target sensitive populations like the less educated and housewives who can fall prey to unnecessary misconceptions and have adverse impact on their quality of life.

Oral cancer phobia is a stark reality and its 100 % occurrence in the OSMF patients in the present study along with increased state and trait anxiety points towards the psychological turmoil these patients might be undergoing. Considering the aim of the present study, CAI was used with the intend to analyse the absence or presence of oral cancer phobia in OSMF patients. Such tools could be used in future studies to evaluate oral cancerphobia and if found to be present then appropriate psychological counselling for it should be considered an important factor in the patient management protocol. This will not only help to motivate patients to undergo treatment, but also visit for regular follow-up appointments. Thus enabling oral cancer phobia assessment during and post management. Also further studies with larger sample size, multicentric & geographical region wise should be conducted to further evaluate the association of various socio-demographic factors with respect to oral cancer phobia in OSMF.

## 5 Conclusion

Oral submucous fibrosis, a chronic fibrosing premalignant condition, undoubtedly affects the quality of life. Nevertheless, additional psychological distresses like cancer phobia and anxiety can lead to further worsening of the condition. Therefore it is recommended to include early identification and redressal of these psychological distresses in the management protocol of OSMF patients.

## Declaration of competing interest

The authors declare that they have no known competing financial interests or personal relationships that could have appeared to influence the work reported in this paper.
